# Neuroprotective effect of the active components of three Chinese herbs on brain iron load in a mouse model of Alzheimer’s disease

**DOI:** 10.3892/etm.2015.2234

**Published:** 2015-01-29

**Authors:** XIAN-HUI DONG, WEI-JUAN GAO, WEI-NA KONG, HONG-LIN XIE, YAN PENG, TIE-MEI SHAO, WEN-GUO YU, XI-QING CHAI

**Affiliations:** 1 Department of Anatomy, Chengde Medical University, Chengde, Hebei 067000, P.R. China; 2 Department of Pathophysiology, Hebei University of Chinese Medicine, Shijiazhuang, Hebei 050200, P.R. China; 3 Bioreactor and Protein Drug Research and Development Center of Hebei Universities, Hebei Chemical and Pharmaceutical College, Shijiazhuang, Hebei 050000, P.R. China

**Keywords:** Alzheimer’s disease, APP/PS1 transgenic mouse, Morris water maze, β-amyloid peptide, divalent metal transporter 1, ferroportin 1

## Abstract

Alzheimer’s disease (AD) is a neurodegenerative brain disorder and the most common cause of dementia. New treatments for AD are required due to its increasing prevalence in aging populations. The present study evaluated the effects of the active components of *Epimedium*, *Astragalus* and *Radix Puerariae* on learning and memory impairment, β-amyloid (Aβ) reduction and brain iron load in an APP_swe_/PS1_ΔE9_ transgenic mouse model of AD. Increasing evidence indicates that a disturbance of normal iron homeostasis may contribute to the pathology of AD. However, the underlying mechanisms resulting in abnormal iron load in the AD brain remain unclear. It has been hypothesized that the brain iron load is influenced by the deregulation of certain proteins associated with brain iron metabolism, including divalent metal transporter 1 (DMT1) and ferroportin 1 (FPN1). The present study investigated the effects of the active components of *Epimedium*, *Astragalus* and *Radix Puerariae* on the expression levels of DMT1 and FPN1. The treatment with the active components reduced cognitive deficits, inhibited Aβ plaque accumulation, reversed Aβ burden and reduced the brain iron load in AD model mice. A significant increase was observed in the levels of DMT1-iron-responsive element (IRE) and DMT1-nonIRE in the hippocampus of the AD mouse brain, which was reduced by treatment with the active components. In addition, the levels of FPN1 were significantly reduced in the hippocampus of the AD mouse brain compared with those of control mice, and these levels were increased following treatment with the active components. Thus, the present study indicated that the active components of *Epimedium*, *Astragalus* and *Radix Puerariae* may exert a neuroprotective effect against AD by reducing iron overload in the AD brain and may provide a novel approach for the development of drugs for the treatment of AD.

## Introduction

Alzheimer’s disease (AD) is a neurodegenerative brain disorder and is the most common cause of dementia. AD is characterized by Aβ plaque accumulation, intracellular tangles, neuronal loss in selective brain regions and a wide range of challenging behavioral disturbances, which may ultimately result in fatality (1).

Previous studies have indicated that iron is involved in the progression of AD (2), and that altered iron homeostasis may be an important factor in the pathogenesis of the disease (3). However, the underlying mechanisms resulting in the abnormal iron load in the AD brain remain unclear. It has been hypothesized that the brain iron load may be affected by the altered expression of certain brain iron metabolism-associated proteins, including divalent metal transporter 1 (DMT1) and ferroportin 1 (FPN1) (4).

DMT1, also known as natural resistance-associated macrophage protein 2 (Nramp2) or divalent cation transporter 1 (DCT1), is a proton-coupled metal ion transport protein, and was the first transmembrane iron transporter to be identified in mammals (5). DMT1 is a widely expressed protein, with 12 putative transmembrane-spanning domains, and is responsible for the uptake of a broad range of divalent metal ions (6), including Fe^2+^, Zn^2+^, Mn^2+^, Co^2+^, Cd^2+^, Cu^2+^, Ni^2+^ and Pb^2+^ (7–9). The four isoforms of DMT1 are distinguished through their mRNA transcripts, which vary at their 5′-UTR and 3′-UTR. Two of these four transcripts contain an IRE at the 3′-end (10,11). Thus, the carboxy-terminal DMT1 protein isoforms are designated -IRE and -nonIRE. FPN1 is the sole exporter of iron and is responsible for iron absorption in the intestines, recycling of erythrocyte iron by macrophages and maternal delivery of iron to the fetus (12,13).

Deferoxamine (DFO) is an iron chelator that significantly alleviates the symptoms of patients with AD, resulting in a notable neuroprotective effect (14,15). However, chemical drugs may exhibit adverse effects, including oral side effects, and are costly, suggesting that research into alternative therapies is required. The use of the active components of *Epimedium*, *Astragalus* and *Radix Puerariae* may circumvent these shortcomings. Furthermore, these compounds may scavenge free radicals, reduce inflammation, adjust multiple viscera functions (16) and reduce brain iron overload (17,18).

Therefore, the present study aimed to investigate the potential role of the active components of *Epimedium, Astragalus* and *Radix Puerariae* on brain iron load in AD. An APP/PS1 transgenic mouse model of AD was established and treated with a mixture of the active component compounds. The Morris water maze test was used to evaluate whether the active component treatment was able to attenuate the cognitive deficits of AD in the mouse model. Following behavioral testing, the Aβ plaque accumulation and brain iron load in the mouse hippocampus were determined. Furthermore, the expression levels of DMT1 and FPN1 were examined to clarify the molecular mechanisms underlying the abnormal brain iron load in AD.

## Materials and methods

### Animals and treatments

The present study was approved by the Ethics Committee of Chengde Medical University (Chengde, China). In total, 30 male APP_swe_/PS1_ΔE9_ (APP/PS1) mice and ten C57BL/6J (C57) mice (both obtained from Beijing HFK Bioscience Co., Ltd., Beijing, China) were used in the present study. The APP/PS1 transgenic mouse model of AD overexpresses the Swedish (K594M/N595L) mutation of APP, with presenilin 1 (PS1) deleted in exon 9 and a C57 genetic background. The APP/PS1 mice were genotyped using polymerase chain reaction (PCR). The mice were housed under 12-h light/dark cycle conditions and were fed and watered regularly. Six-month-old APP/PS1 transgenic mice were divided at random into three groups. The AD model group contained APP/PS1 mice that received no treatment. The active component group consisted of APP/PS1 transgenic mice that received the active components of *Astragalus*, *Radix Puerariae* and *Epimedium* (120, 80 and 80 mg/kg, respectively), which was administered via oral gavage. The DFO group received 30 mg/kg DFO and was used as a positive control. In addition to the these three groups, a fourth (C57) group contained ten male C57 mice and was included as a normal control.

### Preparation of the active component treatment

**Figure 1:**
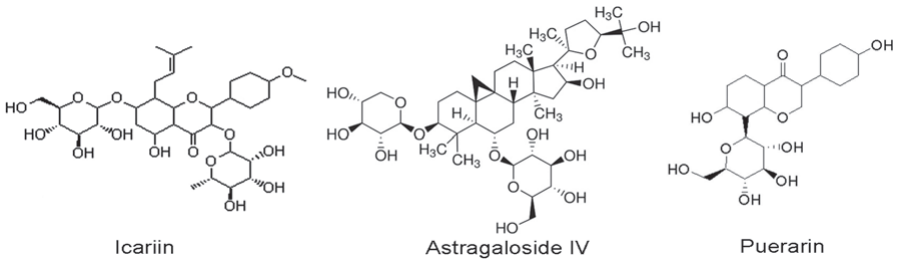
Chemical structures of icariin, astragaloside IV and puerarin.

The treatment comprised of the active components of three Chinese medicinal herbs, *Epimedium*, *Astragalus* and *Radix Puerariae*, including icariin, astragaloside IV and puerarin, respectively (Fig. 1). These substances were purchased from Nanjing Zelang Medical Technology Co., Ltd (Nanjing, China) and were >98% pure. Icariin, astragaloside IV and puerarin were dissolved or suspended in distilled water at a ratio of 3:2:2.

### Morris water maze

Behavioral examinations were conducted in a Morris water maze at week 8 of drug treatment, as described in a previous study (19). Briefly, a circular black pool (diameter, 1.2 m; depth, 55 cm) was filled with water to a depth of 30 cm at 22°C. A clear circular platform (diameter, 10 cm) was submerged 2 cm underwater in the northeast quadrant of the pool. Each mouse underwent four trials per day for six consecutive days. During the place navigation trial, mice were placed randomly into the pool facing the wall individually from four preset starting points, and were allowed to swim for a maximum of 120 sec or until they located the platform. On the sixth day, the spatial probe trial was conducted, in which the platform was removed from the pool and the mice were allowed to swim for 120 sec. The total swim time (escape latency; sec); the number of times the animal crossed the previous location of the platform (platform-crossing); the time that the animal spent in the quadrant where the platform in (time spent in the target quadrant; sec); and the average swimming velocity (m/s) were recorded using a video tracking system (SLY-WMS Morris Water Maze System; Beijing Sunny Instruments Co., Ltd., Beijing, China). The scores of the animal behavior when searching for the platform (search strategies score) were recorded by a researcher blind to the treatment of the mouse. The search strategy categories used were direct swim, tendency search, random search and circle search, which received a score of 1, 2, 3 and 4, respectively.

### Tissue preparation

Following the Morris water maze experiments, the mice were anesthetized with 50 mg/kg sodium pentobarbital (Tianjin Fu Chen Chemical Reagents Factory, Tianjin, China) administered intraperitoneally, then euthanized by decapitation. The brains were removed rapidly and divided into hemispheres on an ice-cooled board. The hippocampus and cerebral cortex were dissected from one hemisphere and stored at −80°C for western blot analysis. The remaining hemisphere was fixed in 4% paraformaldehyde (Tianjin Fu Chen Chemical Reagents Factory) in phosphate-buffered saline (PBS; Tianjin Fu Chen Chemical Reagents Factory) at 4°C overnight. The hemisphere was then embedded in paraffin (Tianjin Fu Chen Chemical Reagents Factory), cut into 5-μm sections and stored at room temperature until required for morphological analysis.

### Immunohistochemistry (IHC) and Aβ load measurement

Standard avidin-biotin complex IHC staining was performed to analyze the distribution of Aβ plaques in the APP/PS1 mouse brain. Briefly, paraffin sections were dewaxed, rehydrated and treated in 0.1 M Tris-hydrogen chloride (HCl) buffer (pH 7.4) containing 3% hydrogen peroxide (H_2_O_2_; Tianjin Fu Chen Chemical Reagents Factory) for 10 min to reduce endogenous peroxidase activity. Following washing with Tris-buffered saline, the sections were boiled in citric acid buffer (Tianjin Fu Chen Chemical Reagents Factory) for 3 min at 800 W in a microwave oven. The sections were then rinsed in running water, treated with 5% bovine serum albumin (Biotin-Streptavidin HRP Detection system, ZSGB-BIO, Beijing, China) for 30 min, and subsequently incubated overnight with monoclonal mouse anti-human Aβ antibody (1:500, #A5213; Sigma-Aldrich, St. Louis, MO, USA) at 4°C. The sections were rinsed in running water and subsequently incubated with biotinylated goat anti-mouse immunoglobulin G (IgG) (1:200; Biotin-Streptavidin HRP Detection system) for 1 h and with streptavidin peroxidase (Biotin-Streptavidin HRP Detection system) for a further 1 h at room temperature. Following rinsing, the sections were stained with 0.025% diaminobenzidine (DAB; Sigma-Aldrich) for 1 min. The stained sections were dehydrated, cleared, covered with neutral balsam and examined under a light microscope equipped with a digital camera (BH-2; Olympus Corporation, Tokyo, Japan). Control group mice were treated with identical solutions but without primary antibody, followed by all subsequent incubations as described above.

### Quantitative image analysis was performed for Aβ IHC with micrographs of five sections per brain

The number of Aβ-positive plaques in the cortex and hippocampus was counted, and comparisons between the control and treatment groups were made. Aβ burden was assessed as the percentage of the total area of the cortex and hippocampus that contained regions of Aβ deposits. The data were analyzed using Image-Pro Plus software, version 6.0 (Media Cybernetics, Inc., Rockville, MD, USA).

### Perls’ reaction

Perls’ reagent, potassium ferrocyanide, reacts with Fe^3+^ in the presence of HCl to form an insoluble pigment known as Prussian blue (20). Following fixation with 4% paraformaldehyde, the brains were stained with Perls’ solution. Paraffin sections were deparaffinized and rehydrated. For the enhancement of iron staining signals, the sections were incubated with 0.75 mg/ml 3,3′-DAB and 0.07% H_2_O_2_ in 1 M Tris-HCl (pH 7.5) for 5 min, followed by rinsing with PBS. The sections were immersed for 2 h in Perls’ blue staining solution, which was prepared immediately prior to use by mixing equal parts of 20% HCl and 20% potassium ferrocyanide (Tianjin Fu Chen Chemical Reagents Factory). Light blue spots were detected using the Perls’ reaction without DAB; however, without DAB enhancement, Perls’ reaction is not very sensitive. Therefore the concentration of the Perls’ liquid was increased and the contrast of the image was altered. The stained sections were dehydrated, cleared, covered with neutral balsam and examined under a light microscope equipped with a digital camera.

### Western blot analysis

The cerebral cortex tissue fragments were minced into small pieces and homogenized in chilled lysis buffer (Tianjin Fu Chen Chemical Reagents Factory) overnight at 4°C. The lysis buffer contained 50 mM Tris-HCl, 150 mM NaCl, 1% Nonidet P-40, 0.25% sodium deoxycholate, 1 mM phenylmethanesulfonylfluoride, 10 mg/ml leupeptin, 1 mM Na_3_VO_4_, 0.1% SDS and 1 mM NaF. Cell pellets were lysed directly on the culture dishes using the lysis buffer. The lysates were collected, centrifuged at 80,000 × g for 30 min and total proteins were quantified using a bicinchoninic acid kit (Multisciences Biotech Co., Ltd., Hangzhou, China). The supernatant was removed, portioned into aliquots and stored at −80°C. A 50-μg sample of each of the total proteins was subjected to SDS-PAGE using 10% gradient Tris/glycine gels (Tianjin Fu Chen Chemical Reagents Factory) and the separated proteins were transferred onto polyvinylidene difluoride membranes (EMD Millipore, Temecula, CA, USA). Subsequent to blocking in 5% nonfat milk for 1 h, the membranes were incubated overnight with the following primary antibodies at 4°C: Polyclonal rabbit anti-rat DMT1-IRE (#NRAMP21-A; 1:3,000) and DMT1-without IRE (#NRAMP23-A; 1:2,000) antibodies from Alpha Diagnostics International Inc., Owings Mills, MD, USA; polyclonal rabbit anti-mouse FPN1 (1:13,000; #MTP11-A; Alpha Diagnostics International Inc., San Antonio, TX, USA) and monoclonal rabbit anti-rat anti-β-actin antibody (1:5,000; Hua Han Biopharmaceutical Holdings Ltd., Shijiazhuang, China). The membranes were washed, then incubated with horseradish peroxidase-conjugated goat anti-rabbit IgG secondary antibody (1:10,000; Kirkegaard & Perry Lab Inc., Gaithersburg, MD, USA) for 2 h. Protein levels were quantified from western blot analyses using a densitometer (DU800; MJ Research, Inc., St. Bruno, QC, Canada), and normalized against β-actin.

Immunoreactive bands were visualized using SuperSignal West Pico Chemiluminescent Substrate (Pierce Biotechnology Inc., Rockford, IL, USA) using ChemiDoc XRS system (Bio-Rad Laboratories, Inc., Hercules, CA, USA) with Image-Pro Plus software, version 6.0.

### Statistical analysis

Data from the water maze escape latency were analyzed using repeated-measures analysis of variance (ANOVA) with the group as the between-subjects independent variable and day of trial as the within-subjects independent variable. Univariate ANOVAs were conducted for single dependent variables in the water maze probe trial and neurochemical assays, with the group as the between-subjects independent variable. Following a significant result in an omnibus ANOVA, Bonferroni post-hoc comparisons were conducted. The comparisons of most interest were the AD model group vs. C57 control group and the drug-treated groups vs. the AD group. All analyses were performed using SPSS software for Windows, version 17.0 (SPSS, Inc., Chicago, IL, USA) and P<0.05 was considered to indicate a statistically significant difference.

## Results

### Morris water maze

**Figure 2:**
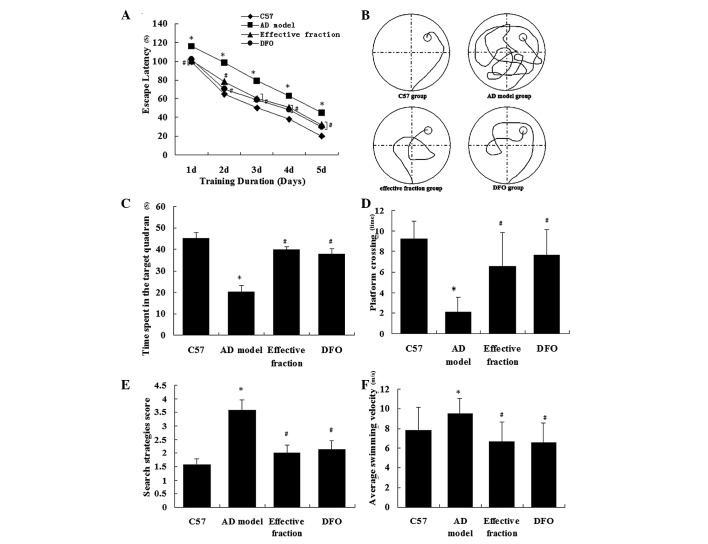
Behavioral performance of animals in the Morris water maze. (A) Average escape latencies. (B) Representative swimming paths on day 5 of the place navigation trial were recorded with a video tracking system. (C) Percentage time spent in the quadrant that previously contained the platform. (D) Search strategies score. (E) Number of crossings to the previous location of the platform. (F) Average swimming velocity during the spatial probe trial. Data are presented as the mean ± standard error (n=10 per group). ^*^P<0.01 vs. C57 and ^#^P<0.01 vs. AD model groups. AD, Alzheimer’s disease; DFO, deferoxamine.

The Morris water maze experiment was conducted to investigate whether the active component treatment was able to reduce cognitive deficits in APP/PS1 mice. The ability of the mice to learn and process spatial information was tested using a Morris water maze. The analysis of the place navigation trial demonstrated that the escape latencies reduced between days 1–5 in all groups (Fig. 2A). The AD model mice exhibited longer escape latencies than the C57 control mice (P<0.01). Mice in the active component and DFO groups displayed significantly reduced escape latencies compared with the AD model mice (P<0.01). There was no significant difference in escape latency between the active component and DFO groups (P>0.79). These results indicate that the active component treatment mitigated the impairment of spatial learning and memory in the AD model mice. Fig. 2B displays the representative swimming paths of mice in the four groups. The analysis of the spatial probe trial indicated that the percentage time spent in the target quadrant (Fig. 2C), the search strategies score (Fig. 2D), the platform-crossing times (Fig. 2E) and the average swimming velocity (Fig. 2F) varied significantly between the groups. The AD model mice spent significantly less time in the quadrant containing the platform than the C57 group mice (P<0.01; Fig. 2C). The search strategies score was reduced in the AD model group compared with the C57 group (P<0.01; Fig. 2D). Furthermore, the number of crossings to the previous location of the platform was reduced in the AD model group compared with the C57 control group (P<0.01; Fig. 2E). The mice in the active component group spent more time in the target quadrant, scored higher in search strategies and exhibited more platform-crossing times than those in the AD model group. These results indicate that the ability of the mice to use spatial cues for the localization of the platform was impaired in the AD model group (P<0.01) and was improved by treatment with the active components of *Astragalus*, *Radix Puerariae* and *Epimedium*. The average swimming velocity was increased in AD model group compared with the C57 group (P<0.01). Mice in the active component and DFO groups exhibited slower average swimming velocities (P<0.01), indicating that the AD model mice did more unnecessary swimming. Collectively, these results suggest that treatment with the active components of *Astragalus*, *Radix Puerariae* and *Epimedium* attenuated the cognitive deficits on learning and memory performance in AD model mice.

### Treatment with the active components of Astragalus, Radix Puerariae and Epimedium inhibits Aβ plaque accumulation and reverses Aβ burden in the brains of APP/PS1 mice

**Figure 3:**
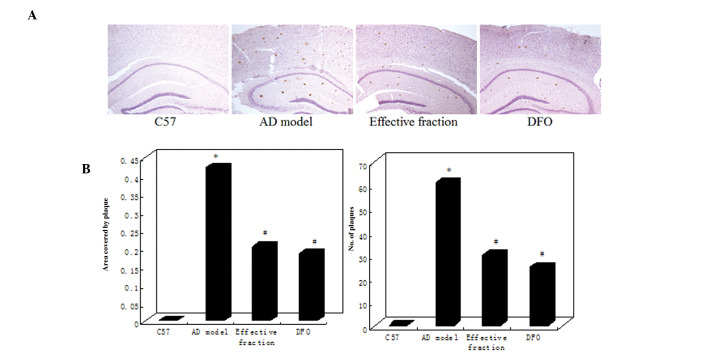
Active component treatment reduces Aβ plaque formation in the APP/PS1 mouse brain. (A) Micrographs of prepared brain tissue display Aβ-positive plaques in the transgenic mouse brain. Fewer Aβ plaques were observed in the active component and DFO group mice compared with the AD model group mice. No Aβ plaques were detected in the C57 group mice. (B) Quantification of Aβ plaques indicated that the number of (left) and area covered by (right) the Aβ plaques were reduced in the active component and DFO group mice compared with the AD model group mice (n=10 per group). ^*^P<0.01 vs. C57 and ^#^P<0.01 vs. AD model groups. AD, Alzheimer’s disease; DFO, deferoxamine; Aβ, β-amyloid.

The effects of the active components of *Astragalus*, *Radix Puerariae* and *Epimedium* on Aβ deposition in the APP/PS1 mouse brain were evaluated using IHC (Fig. 3). The APP/PS1 model mice exhibited a marked increase in the number and size of Aβ-immunoreactive senile plaques in the cortex and hippocampus. However, the active component and DFO group mice displayed a clear reduction in plaque number and Aβ burden compared with the AD model mice (Fig. 3A). Quantitative analysis indicated that the plaque numbers in the brains of the AD model group significantly increased to 62.13% compared with the C57 group (P<0.01; Fig. 3B). The Aβ burden was determined by measuring the areas of Aβ-positive neuritic plaques in the brain. The Aβ plaque area was significantly increased in the AD model group compared with the C57 group (Fig. 3B). However, the brains of active component and DFO group mice exhibited a significant reduction in plaque count and Aβ burden compared with AD model mice. No differences in plaque number or Aβ burden were identified between the active component and DFO groups (Fig. 3B).

### Brain iron deposition was assessed using Perls’ staining

**Figure 4:**
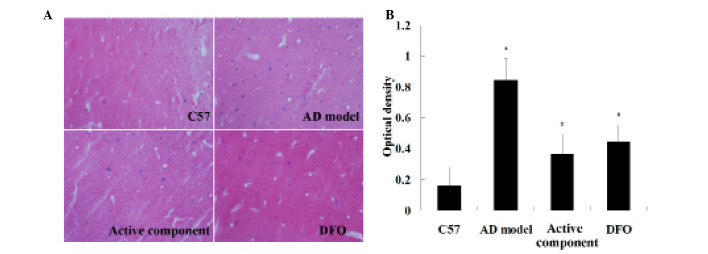
Treatment with the active components reduces the iron load in the APP/PS1 mouse brain. (A) Micrographs of prepared brain tissue display the iron load in the mouse brain. Fewer blue spots were observed in the active component and DFO group mice compared with the AD model group mice. Fewer blue spots were observed in the C57 group mice. (B) Quantification of the blue spots indicated that iron load was reduced in the active component and DFO group mice compared with those in the AD model group mice (n=10 per group). ^*^P<0.01 vs. C57 and ^#^P<0.01 vs. AD model groups. AD, Alzheimer’s disease; DFO, deferoxamine.

Light blue spots were detected using the Perls’ reaction without DAB; however, without DAB enhancement, Perls’ reaction is not very sensitive. Therefore the concentration of the Perls’ liquid was increased and the contrast of the image was altered. The influence of the active components of *Astragalus*, *Radix Puerariae* and *Epimedium* on brain iron load in the APP/PS1 mouse brain was evaluated using Perls’ staining (Fig. 4A). Quantitative analysis indicated that the blue spot number in the brains of the AD model group increased significantly compared with the C57 group (P<0.01; Fig. 4B). The brains of the active component and DFO groups displayed a significant reduction in blue spots compared with the AD model mice. No difference in the number of blue spots was observed between the active component and DFO groups (Fig. 4B). Thus, the AD model group exhibited a marked increase in brain iron load and the active component treatment appeared to reduce this iron load in the AD model mouse brain.

### Western blot analysis

**Figure 5:**
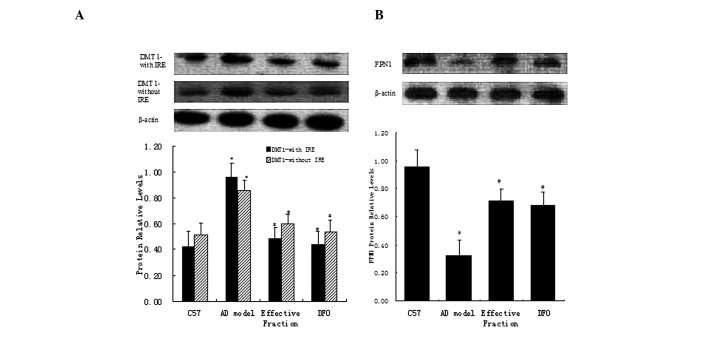
Influence of active component treatment on the protein levels of DMT1 and FPN1. Levels of (A) DMT1-IRE, DMT1-nonIRE and (B) FPN1 (n=10 per group). ^*^P<0.01 vs. C57 and ^#^P<0.01 vs. AD model groups (two-way analysis of variance). DMT1-IRE, divalent metal transporter 1-iron response element; FPN-1, ferroportin-1, AD, Alzheimer’s disease; DFO, deferoxamine.

DMT1 and FPN1 proteins were extracted from the mouse hippocampus in order to quantify the differences in their expression levels between the groups. Western blot analyses for DMT1-IRE and DMT1-nonIRE revealed a clear band at 64 kDa, matching the predicted molecular mass of these proteins. Quantification of the expression levels of DMT1-IRE and DMT1-nonIRE indicated that the levels of the two proteins were significantly higher in the hippocampus of the AD model group than the C57 group, whilst the active component and DFO group mice presented a significant reduction compared with the AD model mice (Fig. 5A). Western blot analysis of FPN1 displayed a major band at 62 kDa, which matched the predicted molecular mass of these proteins. Quantification of the expression levels of FPN1 indicated a significant reduction in the hippocampus of AD model mice compared with the C57 group mice (Fig. 5B), which was partly reversed in the active component and DFO groups.

## Discussion

AD is a neurodegenerative disorder, characterized clinically by progressive memory loss and neuropathologically by extracellular amyloid plaques (21). AD is a typical age-dependent neurodegenerative disease that affects 5% of individuals >65 years, 20% of those >85 years and >33% of those >90 years; >20 million people are affected worldwide (22) and this figure is predicted to rise in the future, due to an increase in the elderly population.

Marked progress has been made in the last 20 years in understanding the pathophysiology of AD (23). However, effective preventative and therapeutic treatments remain to be produced. In the absence of preventative and therapeutic methods, the prevalence of AD will continue to increase as life expectancy increases (24). Therefore, the development of novel treatments for AD is an urgent requirement, in view of the increasingly aged populations (25).

Previous evidence has indicated that a disturbance of normal iron homeostasis may contribute to the pathology of AD, which suggests that iron chelation may be an effective therapeutic intervention (26). The use of an iron-chelating agent, such as DFO, may significantly alleviate symptoms in patients with AD and produce a notable neuroprotective effect. However, these chemical drugs present a number of problems, including adverse effects, such as oral side effects.

Traditional Chinese medicine (TCM) is a complete medical system that has been practiced for >3,000 years. The active component treatment used in the present study was composed of the active components of the TCM herbs *Epimedium*, *Astragalus* and *Radix Puerariae*, including icariin, astragaloside IV and puerarin, respectively. According to TCM theories, these active components may be useful in the treatment of patients with neuropsychiatric disorders. The active components of *Epimedium*, *Astragalus* and *Radix Puerariae* may circumvent the shortcomings of chemical iron-chelators. Furthermore, these compounds may scavenge free radicals, reduce inflammation, adjust the functions of multiple viscera(16) and reduce iron overload of recession in the central nervous system (17,18). The present study aimed to demonstrate these effects and to explain the underlying mechanisms using contemporary methodology and transgenic animal models of AD.

Multiple genetically modified transgenic animal models include APP/PS1/tau (27,28) and Cdk5/P35/tau. APP/PS1/tau mice express human APP695(Swe), PS1(M146V) and Tau (P301L) under the control of the mouse Thy-1 promoter.

In the present study, APP/PS1 mice were used as the AD model. The mice express human APP with the Swedish mutations (K670N/M671L) at the β-secretase cleavage site and PS1 (PS1dE9) (29); with a C57BL/6J background. In addition, C57BL/6J mice were used in the normal control group. APP/PS1 mice have been reported to demonstrate impaired learning and memory in the Morris water maze test (30) and the results of the present study were consistent with this. The neuroprotective effects of the active components of *Epimedium*, *Astragalus* and *Radix Puerariae* were investigated in this mouse model in the current study.

A number of previous studies have demonstrated the neuroprotective effect of metal chelators, which may be clinically applicable in the treatment of AD (31,32). One previous study reported that the iron chelator DFO slowed the clinical progression of cognitive decline associated with AD (3); therefore, the present study utilized DFO as a positive control.

The clinical manifestations of cognitive deficits in the early stages of AD include impaired learning and memory ability. Behavioral data obtained using the Morris water maze in the present study confirmed a significant impairment in learning and memory performance in APP/PS1 mice, which was consistent with previous studies (33,34). Collectively, the escape latency, platform-crossing, time spent in the target quadrant and average swimming velocity results suggested that the active component treatment improved the learning and memory deficits of the AD model mice. These results substantiated the presence of neurotoxicity in the model mouse brain and indicated that the active component treatment was able to attenuate the resulting cognitive deficits associated with AD.

The deposition of Aβ in the brain induces a series of neurotoxic effects and is considered to be the primary factor in the emergence and progression of AD (35,36). The most common form of Aβ is Aβ1–42, located primarily in discrete Aβ deposits. The more soluble Aβ1–40 form is associated with blood vessels (37) and may develop later in the course of the disease (38). The reduction of Aβ levels, particularly the more toxic Aβ1–42 form, is considered to be one of the most important therapeutic targets for the treatment of AD. Prior studies indicate that increased Aβ production in the hippocampus and cortex leads to synaptic impairment, neuronal loss and memory deficits (39,40). Furthermore, synaptic dysfunction has been identified in the associated cortices and hippocampus of the AD brain (41,42). These observations suggest that the excessive production of Aβ peptides in the brain is a pivotal factor in AD pathology. In the present study, Aβ neuropathology was quantified using immunohistochemistry. Treatment with the active components of *Astragalus*, *Radix Puerariae* and *Epimedium* inhibited Aβ plaque accumulation and reversed the Aβ burden in the APP/PS1 mouse brain.

Iron is an essential nutrient involved in numerous vital biological processes, and iron deficiency and overdose are harmful to the health of humans. Therefore, iron homeostasis is a strictly regulated process in healthy individuals. The uptake, use, storage and excretion of iron are maintained in a dynamic balance to ensure that the iron content in the body can fulfill physiological needs without excessive accumulation.

Iron is an intrinsic producer of reactive oxygen species through associated processes including hydroxyl radical formation, glutathione consumption, protein aggregation, lipid peroxidation and nucleic acid modification (43). Factors that disturb iron homeostasis may lead to disease; therefore, iron metabolism has long been the focus and interest of numerous researchers.

Metal ions are implicated in the pathogenesis of AD (44,45) and have been demonstrated to participate in APP expression, Aβ generation and the production of oxidative compounds (46,47). Therefore, it can be hypothesized that metal transporters may serve important functions in the pathogenesis of AD by altering metal homeostasis (48,49). In the present study, the brain iron load was evaluated using Perls’ staining and iron was observed to accumulate in the AD brain. Treatment with the active components of *Astragalus*, *Radix Puerariae* and *Epimedium* was observed to relieve brain iron overload of the AD model mice.

In the previous ten years, considerable progress has been achieved in the study of iron metabolism. Numerous genes and proteins have been associated with iron metabolism, including DMT1 (5), transferrin receptor 2 (50), FPN1 (51,52), hephaestin (53,54) and hepcidin (55,56). These studies and others have improved the understanding of iron metabolism and its regulation, and provide a scientific basis for further characterizing the molecular mechanisms of iron metabolism-associated diseases. It has been indicated that the expression and regulation of these genes is tissue-specific and that their expression may be directly or indirectly affected by the iron status in the body (57).

Cellular iron homeostasis is regulated by the iron transporters DMT1 and FPN1. The primary iron uptake transporter is DMT1, while FPN1 (58) functions essentially as an iron efflux transporter (59). DMT1 is involved in iron uptake, which is widely distributed in mammalian tissues. DMT1 is known to contribute to neurodegeneration in animal models of Parkinson’s disease (60); however, a comprehensive description of DMT1 in the AD pathogenesis has not yet been established.

Notably, a previous study on rat brains demonstrated that the expression of the IRE and non-IRE isoforms of DMT1 increase with age (60) and thus, DMT1 has been hypothesized to be the primary risk factor in AD. In the present study, western blot analysis was used to detect the levels of brain iron load-associated DMT1 and FPN1. The levels of the two isoforms of DMT1 were significantly increased in the AD model hippocampus compared with the healthy C57 control, and the level of FPN1 was significantly reduced. Thus, the results of the present study are essentially consistent with the previous study (11). However, the molecular mechanisms responsible for these effects require further investigation.

The underlying mechanisms that result in an abnormal brain iron load may involve the overexpression of the brain iron metabolism-associated proteins DMT1 and FPN1. These results suggest that DMT1 and FPN1 serve critical functions in the iron-mediated neuropathogenesis of AD and that pharmacological inhibition of DMT1 and FPN1 may provide novel therapeutic strategies for treating AD.

In conclusion, treatment with the active components of *Epimedium*, *Astragalus* and *Radix Puerariae* improved the learning and memory deficits of APP/PS1 mice and reduced the Aβ and iron load in the AD brain. Therefore, the active components of *Epimedium, Astragalus* and *Radix Puerariae* may be clinically applicable for the prevention or treatment of AD.
